# Mapping the Invisible Hand: A Body Model of a Phantom Limb

**DOI:** 10.1177/0956797612441219

**Published:** 2012-07

**Authors:** Matthew R. Longo, Catherine Long, Patrick Haggard

**Affiliations:** 1 Birkbeck, University of London; 2 University College London

After amputation, individuals often have vivid experiences of their absent limb (i.e., a *phantom limb*). Therefore, one’s conscious image of one’s body cannot depend on peripheral input only (Ramachandran & Hirstein, 1998). However, the origin of phantom sensations is hotly debated. Reports of vivid phantoms in the case of congenital absence of the limb show that memory of former body state is not necessary (Brugger et al., 2000). According to one view, phantoms may reflect innate organization of sensorimotor cortices (Melzack, 1990). Alternatively, phantoms could reflect generalization from viewing other people’s bodies (Brugger et al., 2000), a sensorimotor example of the classic theory that understanding oneself follows from understanding the “generalized other” (Mead, 1934, p. 154). Because phantom limbs cannot be stimulated, sensory testing cannot directly compare visual and somatosensory influences on representations of phantom limbs. Consequently, empirical investigation of phantoms is limited.

We recently developed a novel method for constructing maps of body representations (Longo & Haggard, 2010), and that method may clarify the sensory origins of phantoms. The hand is occluded, and participants indicate the perceived locations of fingertips and knuckles. The configuration of perceived locations generates a perceptual hand map. We found that these maps are systematically distorted: The hand is represented as shorter and wider than it actually is. Similar distortions characterize early somatosensory processing (see Longo & Haggard, 2010). Although phantom limbs lack physical substance, they have shape and spatial location, which can be measured using our paradigm. Thus, our method provides a unique way to “image” phantom limbs. In the study reported here, we used this method to study the form of a phantom limb in a case of congenital limb absence.

## Method

C. L. is a 38-year-old woman born without a left arm. She has periodic but distinctive experiences of a stable left phantom hand. We compared maps of her phantom left hand with maps of her intact right hand and with the true shape of her right hand. Initially, C. L.’s phantom hand was mapped using the method we described in previous work (Longo & Haggard, 2010). C. L. used a baton in her right hand to indicate the perceived location of the fingertip and knuckles of each finger on the phantom hand. An overhead camera took photographs of these judged locations. Ten maps (each including one judgment of each landmark in random order) were collected. C. L. reported clear sensations of location of her phantom left hand during the task, which she did not find difficult.

Because C. L. cannot use her left hand to point to landmarks on her intact right hand, we asked her to verbally instruct an experimenter who was naive to the purpose of our study to position the baton, so that we could collect five maps of her intact right hand. Before and after we collected each map, the camera took photographs without the occluder so that we could assess the size and shape of the actual right hand. Finally, so that we could compare right-hand and left-hand maps created using the same response modality, we collected five additional maps of the phantom hand by asking C. L. to verbally report the locations to an experimenter who positioned the baton accordingly. The maps of the phantom left hand that were created by C. L.’s pointing and verbal report were highly similar, so we averaged them for the analyses reported here.

From photographs, pixel coordinates of judged locations were coded off-line. A ruler that was included in the photographs taken without the occluder allowed conversion from pixels to centimeters. Finger length (distance between knuckle and fingertip) and distance between knuckle pairs were measured for each map. For the intact right hand, we calculated the percentage of overestimation of these distances relative to the actual proportions of the hand. For the phantom left hand, we calculated the percentage of overestimation relative to a hypothetical left hand with proportions identical to those of the right hand.

## Results and Discussion

**Fig. 1.**
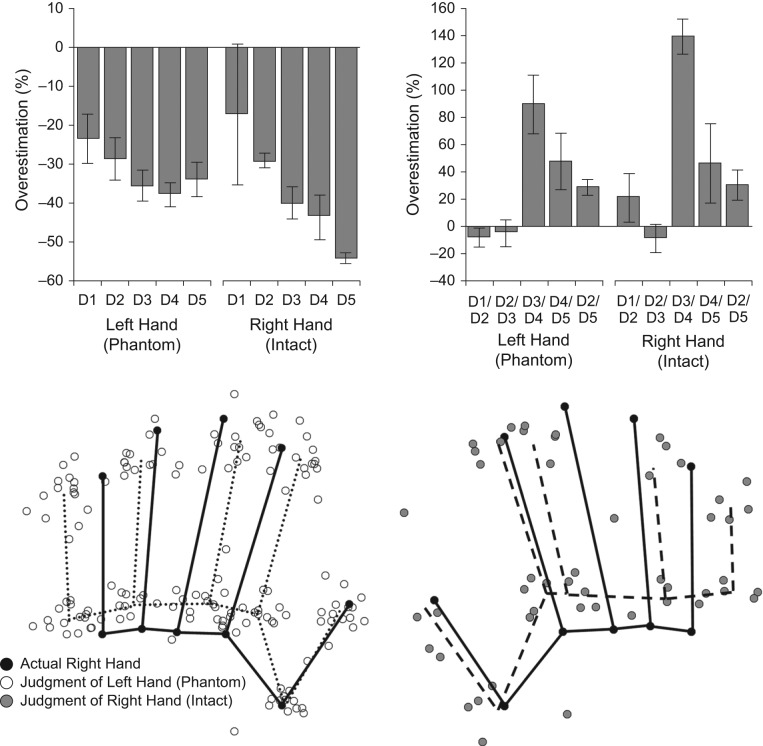
Experimental results. The top row shows the percentage of overestimation of finger length (left) and of spacing between pairs of knuckles (right) for C. L.’s phantom left hand and her intact right hand. “D1” through “D5” refer to the five fingers from the thumb to the little finger, respectively. Values for the absent left hand are based on the actual proportions of the intact right hand. Error bars represent 1 SE. The bottom row shows perceptual maps for C. L.’s phantom left hand (left) and her intact right hand (right) superimposed on her actual hand shape using generalized Procrustes superposition. The actual right hand is mirror reflected for comparison with the phantom left hand.

The top row of Figure 1 shows C. L.’s percentage overestimation of finger length (left panel) and spacing between pairs of knuckles (right panel). The bottom row of the figure shows perceptual maps of C. L.’s intact right hand and of her phantom left hand in Procrustes superposition with the actual shape of her right hand. C. L. showed the pattern of distortions that we found in previous studies (Longo & Haggard, 2010, 2012), both for her intact right hand and for her phantom left hand. First, there was overall underestimation of finger length—phantom left hand: 31.8% underestimation, *t*(14) = −8.99, *p* < .0001; right hand: 36.7% underestimation, *t*(4) = −16.01, *p* < .0001. Second, there was clear overestimation of hand width as measured by the distance between the index- and little-finger knuckles—phantom left hand: 29.0% overestimation, *t*(14) = 5.26, *p* < .0001; right hand: 30.8% overestimation, *t*(4) = 2.80, *p* < .05; this pattern mirrors previously reported anisotropies of tactile receptive fields (Alloway, Rosenthal, & Burton, 1989) and tactile size perception (Longo & Haggard, 2011).

Third, there was a radial-ulnar gradient, with finger-length underestimation increasing from thumb to little finger—phantom left hand: mean β = 2.9% underestimation per finger, *t*(14) = −2.35, *p* < .05; right hand: mean β = 8.9% underestimation per finger, *t*(4) = −2.26, *p* = .087; this pattern mirrors established differences in the sensitivity and size of the cortical territory representing the five fingers (Duncan & Boynton, 2007). Crucially, these distortions were virtually identical for the phantom and intact hands and were independent of the method used to collect the maps. Finally, we investigated the precision of the representation of C. L.’s intact and phantom hands by calculating the variable error for each landmark as the average distance between each judgment and the center of mass of all judgments of that landmark within each block of trials. Across landmarks, variable error was similar for C. L.’s phantom hand (1.21 mm) and her intact hand (1.78 mm).

If phantom hands arise through viewing other people’s limbs, they should correspond to the shape of actual hands. C. L.’s phantom representation did not closely resemble either her own intact right hand or any hand she is likely to have observed in others. Instead, the representation of the phantom, like that of the intact hand, was profoundly distorted in ways that appear to reflect the organization of somatosensory cortex, but are not consistent with visual learning about body form. Our findings are consistent with an innate organization of mental body representations (Melzack, 1990). Our data cannot exclude the possibility that a representation of the intact right hand in the contralateral hemisphere generates the somatosensory organization of the phantom transcallosally. However, reports of phantoms in individuals born without both arms suggest that transcallosal transfer is not necessary for experiencing a phantom (Brugger et al., 2000). By measuring the form and structure of a phantom limb for the first time, we showed that phantoms are not merely simulacra of actual limbs, but instead reflect enduring sensorimotor structures in the brain.

Bodily illusions show that somatosensory afference contributes to bodily awareness (Lackner, 1988), yet is readily overridden by vision (Botvinick & Cohen, 1998). Our results suggest that representation of body structure can exist without either visual or somatosensory input. Such representations nevertheless have a characteristic structure aligned with organizing principles of the somatosensory system. The feeling of embodiment arises not only from interactions with the environment, but also from a basic, and possibly innate, organization of the “body in the brain.”
